# Antisense Oligonucleotide-Mediated Downregulation of IGFBPs Enhances IGF-1 Signaling

**DOI:** 10.3233/JND-230118

**Published:** 2024-03-05

**Authors:** Alper Yavas, Maaike van Putten, Annemieke Aartsma-Rus

**Affiliations:** Department of Human Genetics, Leiden University Medical Center, Leiden, The Netherlands

**Keywords:** Insulin-like growth factor-1, Duchenne muscular dystrophy, antisense oligonucleotides, exon skipping, IGF-binding proteins

## Abstract

Insulin-like growth factor-1 (IGF-1) has been considered as a therapeutic agent for muscle wasting conditions including Duchenne muscular dystrophy as it stimulates muscle regeneration, growth and function. Several preclinical and clinical studies have been conducted to show the therapeutic potential of IGF-1, however, delivery issues, short half-life and isoform complexity have impose challenges. Antisense oligonucleotides (AONs) are able to downregulate target proteins by interfering with their transcripts. Here, we investigated the feasibility of enhancing IGF-1 signaling by downregulation of IGF-binding proteins. We observed that out of frame exon skipping of *Igfbp1* and *Igfbp3* downregulated their protein expression, which increased Akt phosphorylation on the downstream IGF-1 signaling *in vitro*. 3’RNA sequencing analysis revealed the related transcriptome in C2C12 cells in response to IGFBP3 downregulation. The AONs did however not induce any exon skipping or protein knockdown in *mdx* mice after 6 weeks of systemic treatment. We conclude that IGFBP downregulation could be a good strategy to increase IGF-1 signaling but alternative tools are needed for efficient delivery and knockdown *in vivo*.

## INTRODUCTION

Duchenne muscular dystrophy (DMD) is an X-linked, progressive neuromuscular disorder affecting 1 in 5000 to 6000 boys [1]. Diagnosis generally occurs around 2-3 years of age when patients demonstrate early symptoms of the disease including difficulties with climbing stairs, waddling gait and frequent falls. This is followed by loss of ambulation by 10–12 years of age and, even with optimal care, premature death between 20 and 40 years of age [1–3]. The disease is caused by mutations in the *DMD* gene that prevent production of functional dystrophin protein [4]. Dystrophin connects the actin cytoskeleton to the extracellular matrix through the dystrophin-associated protein complex (DGC), and acts as a shock absorber during contractions. In the absence of dystrophin the DGC is disrupted making muscle fibers more susceptible to contraction induced muscle damage [5]. The main pathological characteristics of a dystrophic muscle include unequal diameter of muscle fibers, necrosis, chronic inflammation and fibrosis resulting in replacement of muscle fibers with fibrotic and adipose tissue [6].

There are several genetic-based therapies in use or under investigation addressing the primary *DMD* gene defect, such as viral vector mediated delivery of micro-dystrophin, antisense oligonucleotide mediated exon skipping and stop-codon read through compounds [7]. However, it is challenging to deliver these compounds at sufficiently high dosages without causing an immune response and/or toxicity [8]. Further, the latter two strategies can only be applied to a specific subset of patients based on their genetic mutations [9]. Therefore, investigation of therapeutic approaches to improve disease pathology independently or together with genetic-based therapies has gained attention. Growing knowledge on disease pathology has revealed several downstream targets that could be modulated to slow down disease progression by targeting the secondary defects including muscle loss, impaired muscle regeneration, fibrosis, and inflammation.

Insulin-like growth factor-1 (IGF-1) is one of the regulators of skeletal muscle growth and maintenance through its role in muscle regeneration, cell proliferation, differentiation, muscle protein synthesis and inhibition of protein degradation pathways [10]. In mammals, IGF-1 is mainly synthesized in liver, acting as a systemic growth hormone, but it is also locally produced in extrahepatic tissues including skeletal muscle acting in an autocrine/paracrine manner [11]. Several preclinical studies have shown the effect of IGF-1 on muscle growth and strength development with reduced fibrosis/ inflammation and increased muscle stem cell proliferation capacity [12–14]. Promising preclinical results led to a clinical trial where recombinant IGF-1 (rhIGF-1, Increlex, an FDA-approved drug for growth failure) was administered subcutaneously in glucocorticoid-treated DMD boys for 6 months. This resulted in improved linear growth but did not change the motor function [15].

Most of the IGF-1 actions are mediated by IGF-1 receptor IGF-1R, a transmembrane protein with tyrosine kinase activity that activates PI3K/Akt and MAPK/ERK pathways. The activity of IGF-1 is regulated by IGF-binding proteins (IGFBPs) both in circulation and in the extracellular matrix [16]. IGFBP3 is the most abundant IGFBP by making up 75% of all binding proteins [17]. In circulation, the majority of IGF-1 is found as IGF-1/IFGBP complexes, where about 80% of IGF-1 is present as a 150 kDa ternary complex with the additional binding of acid labile subunit to IGFBP3. This complex cannot exit the circulation due to its huge size and acts as long-term reservoir of IGF-1 by prolonging its half-life [18]. The remaining 20% of IGF-1 is found as binary complexes, IGF-1/IGFBP or free IGF-1 (less than 1%), which can easily cross the circulation and therefore are available to other tissues when needed [19]. In addition to IGFBPs in circulation, which are mostly produced by liver, most IGFBPs are also expressed in extrahepatic tissues, where they act as autocrine/paracrine regulators of IGF-1 bioavailability. In most cases, IGFBPs inhibit IGF-1 actions by preventing its interaction to IGF-1R [20]. For example, relatively low muscle mass in older women is associated with high IGFBP1 levels [21]. Another study showed that elevated IGFBP3 in pancreatic cancer cells contributes to muscle wasting by impairing myogenesis and promoting protein degradation pathways in myotubes in an IGF-1 dependent and independent manner respectively [22]. On the other hand, IGFBPs can potentiate IGF-1 actions, but this mostly relies on decreased binding affinity to IGF-1. Mechanisms that lead to this include cell association by binding to glycosaminoglycans in the cell membrane and/or adjacent extracellular matrix, decreased phosphorylation (IGFBP1) and proteolytic cleavage [23].

Different strategies have been employed to increase IGF-1 signaling in different physiological and pathological conditions. These include systemic administration of rhIGF-1, muscle specific IGF-1 expression via vector induced cDNA insertion, pEGylated IGF-1 administration to escape IGFBPs and administration of different splice isoforms [24]. One of the important factors that limits the potential benefits of these studies is the excess amount of IGFBPs present in circulation that capture the administered IGF-1. Moreover, none of these approaches are able to mimic the complexity of IGF-1 post-transcriptional events and post-translational regulations. Therefore, more intrinsic strategies are needed to make use of IGF-1 signaling in skeletal muscle. Given that IGFBPs restrict IGF-1 bioavailability, both in circulation as ternary complex or by preventing its binding to IGF-1R in the extracellular matrix, downregulating IGFBPs would be a good strategy to enhance IGF-1 signaling.

Antisense oligonucleotides (AONs) are single stranded DNA-like molecules ranging from 18 to 30 nucleotides in length, which can interfere with pre-mRNA and/or mRNA processing via hybridizing in a sequence-specific manner [25]. One of these approaches is splice modulation involving the use of AONs that can bind to a complementary region on the pre-mRNA, thereby creating a steric block. This interferes with the normal protein:RNA or RNA:RNA interactions involved in exon recognition by the splicing machinery [26]. Splice-switching AONs are resistant to RNase-H-mediated mRNA degradation due to sugar modifications. Several AONs resulting in exon inclusion or exon skipping to restore production of a missing protein have received regulatory approval [27, 28]. Other than protein restoration, exon skipping AONs can also be used for gene knockdown when an out of frame exon is skipped from a target transcript [29, 30].

In this study, we downregulated IGFBP1 and IGFBP3 protein levels using the AON-mediated exon skipping approach to reduce production of IGFBP1 and IGFBP3 in order to increase IGF-1 bioavailability and therefore its signaling. Our AONs were successful in inducing targeted exon skipping, which resulted in protein knockdown and increased Akt phosphorylation on the downstream signaling in mouse C2C12 and mIMCD3 cells. The 3’RNA sequencing analysis revealed the affected pathways related to myogenesis in response to IGFBP3 downregulation. On the other hand, the AONs did not result in exon skipping in *mdx* mice pointing out the need of more efficient delivery methods and alternative RNA therapeutics.

## MATERIALS AND METHODS

### Selection of target exons and AON design

Because exon 2 is an out of frame exon in both *Igfbp1* and *Igfbp3* genes, it was selected as the target exon to be skipped by AONs. The RNA sequences of exon2 of both transcripts were downloaded from Ensembl (http://www.ensembl.org/index.html) with 50 nt intronic regions flanking on both sides. The secondary structures with ss count table were obtained using the RNA folding form (mfold) [31]. The same sequences were uploaded to Human Splice Finder 3.0 to determine the potential ESE and ESS motifs on the exons. For each gene, partly open and ESE-rich regions were selected for AON design. Other requirements of the AONs, included self-dimerization, dimerization with each other, binding energy, Tm and GC% content, were analyzed using RNA Structure 6.0 software. All the AONs used in the study were produced by Eurogentec, Belgium with 2’-O-methyl phosphorothioate (2OMePS) modifications (Table 1).

**Table 1:** The AONs used in the study

AON name	Target species	Chemistry	Sequence 5’-3’
Igfbp1AON	Mouse	2OMePS	CUUCCAUUUCUUGAGGUCGG
Igfbp3AON	Mouse	2OMePS	AACUUGGAAUCGGUCACUCG
controlAON	Mouse	2OMePS	UUCAAGUUUAUCUUGCUCUUC

### Cell cultures and AON transfections

For Igfbp3AON transfection, mouse myoblasts C2C12 (American Type Culture Collection ATCC, Manassas, VA, USA) were initiated in proliferation medium containing DMEM with 10% fetal bovine serum (FBS), 1% glucose, and 2% Glutamax (Thermo Fisher Scientific, Waltham, MA, USA) at 37°C with 5% CO_2_. When the cells were about 80% confluent, they were passaged into 6-well plates and incubated in proliferation medium. At full confluency the medium was replaced by differentiation medium containing DMEM with 2% FBS, 1% glucose, and 2% Glutamax. For transfection at day 5 in differentiation media, 2OMePS AONs were diluted to a concentration of 200μM and mixed with an appropriate amount of lipofectamine2000 (Thermo Fisher Scientific, Waltham, MA, USA) in plain DMEM media (6μL per well). After 20 min incubation at room temperature, the AON-lipofectamine mixture was added to each well with different final AON concentrations; 100 nM, 200 nM and 500 nM. As a control, a non-targeting AON was used. After 3-4 h, transfection media was replaced by fresh differentiation media and the cells were left in culture until harvesting time. To track myoblast differentiation along with IGFBP3 downregulation, the cells were transfected with 200 nM AON at day 0 in differentiation media using 2μL lipofectamine2000 per well. Cell morphology was visually inspected by light microscopy at day 1-3-5-7 by taking three random pictures per well.

For Igfbp1AON transfection, mouse kidney cells, mIMCD-3 (ATCC, Manassas, VA, USA) cells were used as the cell line was available in our department with an optimized transfection protocol. The cells were initiated in DMEM F-12 medium (Thermo Fisher Scientific, Waltham, MA, USA) and passaged into 6-well plates when they were 80% confluent. When they reached 70–80% confluency, the same transfection protocol was applied using a lower amount of lipofectamine2000 (2μL per well). The cells were harvested after 24h and 48h for RNA and protein analyses. All transfection experiments were performed with three biological replicates.

### RNA isolation, RT-PCR and qPCR

Transfected cells were harvested at 24h and 48h post transfection for RNA and sequencing and analyses. For myoblast differentiation assay, day 0 transfected cells were harvested at day 1 and 3 for RNA isolation. Total RNA was isolated using TRIsure^TM^ RNA isolation reagent (Bioline, USA). cDNA was synthesized with N6 primers and Tetro reverse transcriptase enzyme (Bioline, USA) using 500 ng of the total RNA. To assess exon 2 skipping in *Igfbp1* and *Igfbp3*, nested PCR was performed. In the first round (20 cycles), 3μl of cDNA was used as template and amplified with the outer primers. In the second round (35 cycles), 1μl of PCR product from the first round was amplified with the inner primers (Table 2). The PCR products were run on 1.5% agarose gels using a 100 bp DNA ladder (Thermo Fischer Scientific, MA, USA). Quantitative PCR (qPCR) experiments, were performed in triplicate per biological samples (x3) with the primers described in Table 2 and SensiMix reagents (GCBiotech, Cambridge, UK) in LightCycler 480. *Gapdh* and *Actb* were used as housekeeping genes. Data analysis was done using LightCycler 480 SW 1.5.1 software (Roche Diagnostics) and LinReg qPCR method.

**Table 2:** Primers used in the study

Genes	Experiment	Sequence 5’-3’
*Igfbp1*	Nested PCR	GTCCTTCCAGATTGGCGTGG GTTGGGCTGCAGCTAATCTC
*Igfbp3*	Nested PCR	GGAGCAGTACCCGCTGAG CACAGTTTGGGATGTGGACG
*Igfbp1*	Nested PCR (outer)/qPCR	CCTCAAGAAATGGAAGGAGCCC TCATCTCCTGCTTTCTGTTGGG
*Igfbp3*	Nested PCR (outer)/qPCR	GACAGAATACGGTCCCTGCC CCCTTCTTGTCACAGTTTGGG
*Gapdh*	qPCR	TCCATGACAACTTTGGCATTG TCACGCCACAGCTTTCCA
*Actb*	qPCR	AAGGCCAACCGTGAAAAGAT GTGGTACGACCAGAGGCATAC

### Western blot

For protein analysis, C2C12 cells and miCD3 cells were harvested 48 h after the transfection (2-3-4-5-6 days after the transfection at day 0 for the myoblast differentiation assay), using 500μl RIPA lysis buffer, and incubated 30 min on ice for complete lysis. For tissue samples, 1 ml RIPA buffer was used for ∼50 mg of tissue. After 20 min centrifugation at 13000 rpm, protein lysates were aliquoted to fresh eppendorf tubes for further analysis. In total, 50μg of protein was mixed up with Laemmli buffer and denatured at 95°C for 5 min followed by 1 h running on 4–20% TGX precast gel (BIO-RAD, USA) at 160 V. Once the gel cassette was dismantled, the proteins were transferred to a 0.2μm nitrocellulose membrane (BIO-RAD, USA) using the Trans Blot Turbo system (BIO-RAD, USA). The membrane was blocked with milk powder dissolved as 5% in TBS for 1 h and treated overnight with the primary antibodies directed towards; IGFBP1 (1:500, PA5-79447), IGFBP3 (1:500, PA5-79452), (Invitrogen, USA) phospho-p44/42 (Erk1/2, 1:3000, #9101), p44/42 (Erk1/2, 1:2000, #9102), phospho-Akt (D9E) XP^®^ (1:3000, #4060), Akt (pan, C67E7, 1:3000, #4691) (Cell Signaling Technology, USA) and GAPDH MAB374 (1:2000, Merck, USA). The next day, the membranes were washed with TBS-T three times for 10 minutes and incubated with the following secondary antibodies in TBS-T for 1h at room temperature; against the source organisms of the primary antibodies IRDye^®^ 680RD Donkey anti-Goat (1:10000), IRDye^®^ 680RD Donkey anti-Rabbit (1:10000), IRDye^®^ 680RD Goat anti-Mouse (1:10000). After three washing steps in TBS-T, the membranes were visualized and analyzed on Odyssey^®^ CLx imaging system (LI-COR Biosciences, Miami USA).

### RNA sequencing

For RNA-seq experiments isolated RNA from 3 biological replicates (3 wells) was further purified using the NucleoSpin RNA purification kit (Macharey-Nagel, Germany). RNA integrity was checked using an Agilent 2100 Bioanalyzer according to the manufacturer’s specifications. The 3’library preparation was done using oligo dT primers which was followed by NovaSeq6000 Illumina sequencing at GenomeScan, Leiden. The RNA-seq analysis was done with the nf-core RNAseq pipeline (v.3.11.1). Briefly, the adapter sequences, bases with low-quality scores, poly(A) tails, primers and other unwanted sequences were trimmed using CatAdapt tool [32]. After trimming, FastQC was used to remove low quality bases (Pred score < 33). The trimmed reads were aligned with the STAR alignment tool (v.2.7.3) against the mouse reference genome (GRCm38/mm10).

### Differential gene expression and pathway analysis

The aligned reads were converted to Binary Alignments Map (BAM) files. Then, the feature Counts tool was used to quantify each gene’s feature from each BAM file [33], producing a final gene count matrix per sample. This data matrix was used as input for quality control analysis in the iDEP.91 [33] environment and also for differential gene (DE) expression statistical analysis with Dseq2 [34] in the Rstudio to plot DE genes in volcano plots. The raw data has been made available on the NCBI database in the BioProject (PRJNA973858). SAMtools, sorting and indexing were performed to prepare the BAM files for visualization on the UCSC genome bowser. The number of reads that map within a gene was determined for each sample for DE expression analysis. The analysis was performed on genes for which the average CPM was above 5. Hereto, we used an R package that contains the R-Shiny application (LUMC/dgeAnalysis v1.5.2) with a Docker image. Once the application initialized in a web browser, the meta data file and the count table that contains the sample information and raw gene counts, respectively, were uploaded and the analyses were performed using Limma/Voom with the following cutoff values for *P*-value = 0.05, log2CPM = 1. The DE genes were used in enrichment analysis to unravel the related pathways and network using GO and KEGG databases.

### Assessment of the compounds in mdx mice

Male *mdx* (C57BL/10ScSn-*Dmd*^*mdx*^/J) mice were bred at the animal facility of the Leiden University Medical Center. Mice were housed in individually ventilated cages, at 20.5°C with 12-hour dark/light cycles. They had *ad libitum* access to standard RM3 chow (SDS, Essex, United Kingdom) and water. All experiments were approved by and performed following the guidelines of the Animal Experiment Committee of the Leiden University Medical Center. Care was taken to limit the burden and distress for the animals as much as possible.

The most optimal AONs for *Igfbp1* and *Igfbp3* exon skipping identified by *in vitro* analysis, were tested in *n* = 3 *mdx* males for 6 weeks starting from 4 weeks of age. For *Igfbp1* and *Igfbp3*, respectively, 20 mg/kg and 40 mg/kg dosages were administrated subcutaneously twice a week during the treatment. Two *mdx* males received 40 mg/kg non-targeting control AON. Blood collection was performed via tail incision method at the start and end of the treatment. The serum was collected after 10 min centrifugation at 3000 rpm. The mice were sacrificed 4 days after the last injection via cervical dislocation. The muscles, liver and kidney were isolated and prepared for RNA and protein analyses. After analysis revealed no exon skipping levels for these samples, we included another cohort of *n* = 3 *mdx* males, aged four weeks, which were subcutaneously treated with 50 mg/kg Igfbp1 or 60 mg/kg Igfbp3 AONs for four times a week for a duration of six weeks. Before sacrifice, blood samples were taken through a small cut in the tail vein and collected in Heparin-coated microvettes (Sarstedt, Nümbrecht, Germany). Plasma was obtained after centrifuging at 3000 x g for 5 min at 4°C. The following markers for liver and kidney damage were measured using Reflotron strips and the Reflotron Sprint system (Roche Diagnostics); urea, glutamic oxaloacetic transaminase (GOT, also known as aspartate transaminase), glutamate pyruvate transaminase (GPT, also known as alanine aminotransferase), and alkaline phosphatase (ALP). Mice were sacrificed 4 days after the last injection and muscles, liver and kidney were isolated and used for RNA and protein analyses.

### Testing of AON self-dimerization and degradation

Self-dimerization and degradation tests were performed by treating the AONs with the mouse serum which was collected during the first *in vivo* experiments. Briefly, 2μl of AONs from the stock solution (500μM) was mixed with 10μL of mouse serum and incubated at 37°C for 24h or 72h. After incubation, the mixtures were run on a 2% agarose gel to visualize the possible dimerization or degradation of the AONs.

### Statistical analysis

Data analysis was performed with GraphPad Prism9.3.1 software (GraphPad, San Diego, CA, USA) and expressed as means±SEM. “n” refers to the number of samples per group. A *P*-value of < 0.05 was considered significant.

IGFBP3 expression on RNA and protein levels during C2C12 myoblast differentiation was assessed by one-way analysis of variance (ANOVA) followed by Bartlett’s correction and Tukey’s multiple comparison. AON-mediated downregulation and the effects on the downstream signaling *in vitro* were analyzed with a paired Student’s *t*-test (two-tailed). The IGFBP3 downregulation throughout myoblast differentiation was assessed with a Repeated-measures (RM) two-way ANOVA (Geisser-Greenhouse corrected) to compare treatment and control groups at each timepoint, followed by Sidak’s multiple comparisons.

“n” refers to biological replicates as indicated and biological replicates consist of technical triplicates in qPCR experiments. Significance levels were set at ^*^p < 0.05, ^**^p < 0.01, ^***^p < 0.001, and ^****^p < 0.0001.

## RESULTS

### IGFBP3 levels during C2C12 cell differentiation

**Fig. 1:**
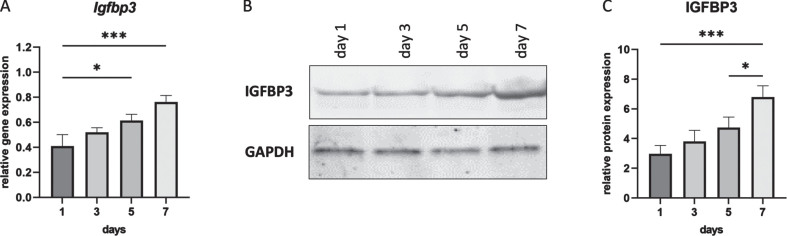
IGFBP3 expression levels during C2C12 cell differentiation. A. *Igfbp3* expression levels detected by qPCR (relative to *Gapdh* and *Actb* reference genes). B. Western blot analysis of IGFBP3 protein levels with GAPDH used as loading control. C. Quantification of the Western blot data. All blots are representative of three independent experiments. *n* =  3 replicates, ^*^p < 0.05, ^***^p < 0.001, one-way ANOVA (followed by Sidak’s multiple comparison) comparing RNA and protein expression levels between each timepoint during differentiation. All data are plotted as means±SEM.

IGFBP1 is not expressed in muscle and indeed we were unable to detect *Igfbp1* expression in cultured mouse myotubes (C2C12)(data not shown), while we could detect *Igfbp3* (Fig. 1). It is known that IGFBP3 has an inhibitory effect on cell proliferation [35]. To observe the expression profile of IGFBP3 during C2C12 cell differentiation, cells were harvested at day 1, 3, 5 and 7. Total RNA and protein were isolated for further analyses. Both qPCR and Western blot results showed the continuous increase of IGFBP3 expression during cell differentiation from day 1 to day 7 in differentiation medium (Fig. 1).

### Exon skipping for Igfbp1 and Igfbp3

**Fig. 2:**
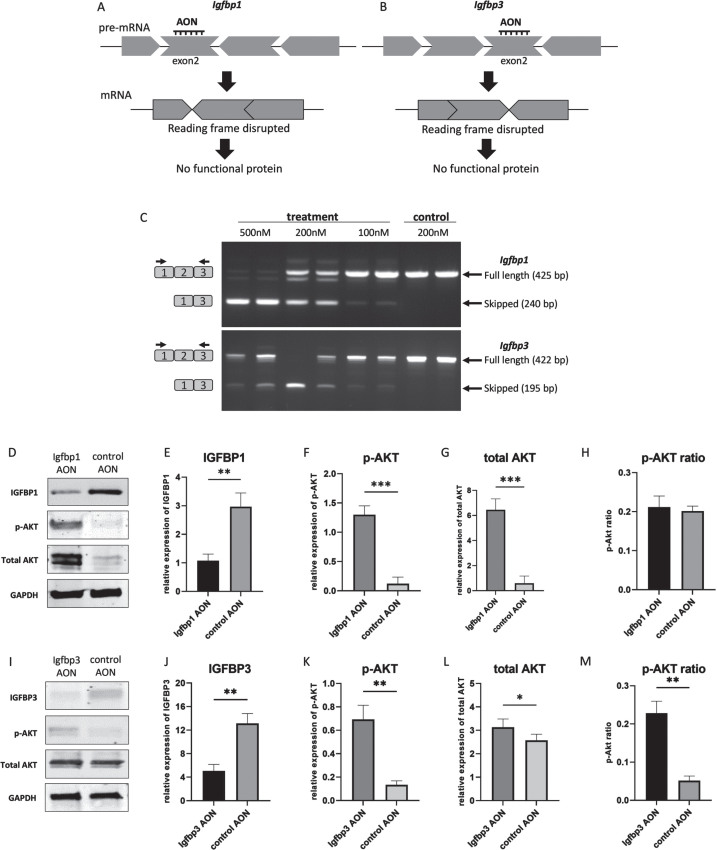
AON-mediated exon skipping to downregulate IGFBP1 and IGFBP3 in miMCD3 and C2C12 cells respectively. A,B. AON-mediated exon skipping approach for IGFBP1 and IGFBP3 knockdown. C. Agarose gel pictures of the RT-PCRs to assess exon 2 skipping in *Igfbp1* and *Igfbp3* genes using two replicates. Upper bands correspond to the wild type products (425 bp, 422 bp) while the lower bands (240 bp, 195 bp) represent the skipped products of *Igfbp1* and *Igfbp3* respectively. Black arrows on the left represent the forward and reverse primers for the detection of the wild type and the skipped products. D,I. Western blot picture showing the effect of exon skipping on IGFBP1 and IGFBP3 as well as on the downstream signaling by assessing phosphorylated AKT, and total AKT levels. E,F,G,H,. Bar graphs showing the densitometry analyses of IGFBP1, p-AKT, total AKT levels with p-AKT ratio upon IGFBP1 downregulation. J,K,L,M. Bar graphs showing the densitometry analyses of IGFBP3, p-AKT, total AKT levels with p-AKT ratio in response to IGFBP3 downregulation. GAPDH was used as loading control. All blots are representative of three independent experiments. *n* =  3 replicates, ^*^p <  0.05, ^**^p < 0.01, ^***^p < 0.001, paired Student’s *t*-test (two tailed) comparing protein levels measured by WB in treated and control groups. All data are plotted as means±SEM.

An AON-mediated exon skipping approach was developed to downregulate IGFBP1 and IGFBP3 proteins. For both *Igfbp1* and *Igfbp3* exon 2 was selected as a target exon to be skipped as they are out of frame exons and when skipped the reading frame would be disrupted, resulting in abolishment of protein synthesis (Fig. 2AB). For the transfection experiments, three different concentrations (100 nM, 200 nM and 500 nM) were used for each AON. Igfbp1AON was tested in mIMCD-3 cells while Igfbp3AON was tested in C2C12 cells. No adverse effect on cell morphology was observed in AON treated cells compared to their untreated counterparts 24h after transfections. For the assessment of the exon skipping in *Igfbp1* and *Igfbp3*, forward and reverse primers were designed to target the flanking exons thereby allowing detection of both wild type and skipped products in one nested PCR reaction (Fig. 2C). *Igfbp1* exon 2 skipping was more efficient and consistent than that of *Igfbp3* in a dose dependent manner. Using 500 nM of AON did not improve exon 2 skipping in *Igfbp3* compared to 200 nM of AON. No skipping was observed in cells treated with the control AON.

### The effect of exon skipping on protein level and downstream pathways

Next, we studied whether AON treatment indeed reduced protein levels. Western blot analyses of proteins isolated 48h after the transfections showed that skipping of exon 2 in *Igfbp1* and *Igfbp3* resulted in a downregulation on protein level compared to control AON treated cells. We also assessed the effect of IGFBP1 and IGFBP3 downregulation on the downstream signaling of IGF-1. For this, Akt phosphorylation, one of the main components of the IGF-1 signaling pathway, was evaluated by Western blot. After densitometry analysis, IGFBP1 expression appeared 3 times lower in AON treated mIMCD3 cells than in control AON treated cells (Fig. 2DE), while IGFBP3 level was ∼2.5 times lower in AON treated C2C12 cells compared to control AON treated cells (Fig. 2IJ). In IGFBP1 downregulated cells, both p-AKT and total AKT were significantly increased (Fig. 2DFG), whereas only p-AKT was increased in IGFBP3 downregulated cells with stable total AKT (Fig. 2IKL). Based on densitometry analysis, AKT phosphorylation was 4 times higher in Igfbp3AON treated cells (Fig. 2M). The AKT phosphorylation in IGFBP1 downregulated cells did not increase significantly due to the increased total AKT level (Fig. 2H). Most likely explanation would be that increased IGF-1 signaling in response to IGFBP1 downregulation in these cells might have affected cell proliferation and thereby influenced p-AKT and total AKT levels. Increased total AKT levels could also be due to a compensatory mechanism activated by the AON transfection. Taken together, our results show that AONs can be used to initiate exon skipping for IGFBP1 and IGFBP3 downregulation *in vitro*. Also, downstream protein analysis suggests that downregulation of IGFBP1 and IGFBP3 enhanced IGF-1 signaling presumably via increasing free-IGF-1 levels.

### Myoblast differentiation dynamics in response to IGFBP3 downregulation

**Fig. 3:**
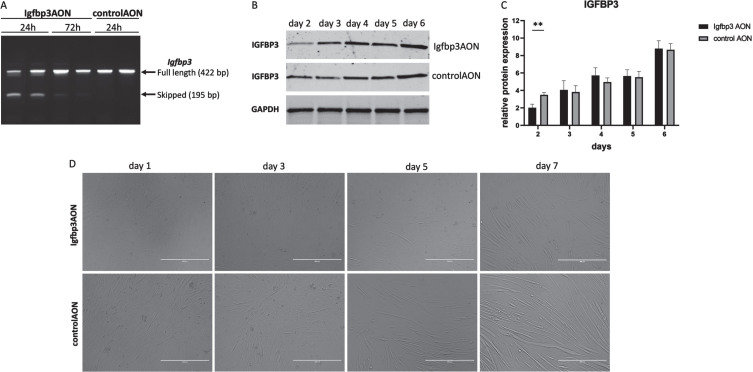
The effect of IGFBP3 downregulation on C2C12 myoblast differentiation. A. Agarose gel picture showing the RT-PCR results of *Igfbp3* exon 2 skipping 24h and 72h after the transfection. The upper bands (422 bp) represent the full length PCR products while the lower bands (195 bp) present the skipped PCR products. B. Western blots showing the IGFBP3 levels in Igfbp3AON treated cells and controlAON treated cells at day 2-3-4-5-6. GAPDH used as loading control. C. Bar graphs showing the densitometry analyses of IGFBP3 downregulation during C2C12 myoblast differentiation. The blots are representative of three independent experiments. D. Pictures showing the cell morphologies during the differentiation in Igfbp3AON treated and controlAON treated C2C12 cells at day 1-3-5-7. (scale: 400μm). *n* =  3 replicates, ^**^p < 0.01, RM two-way ANOVA (followed by Sidak’s multiple comparison) comparing IGFBP3 levels measured by WB at different timepoints during differentiation in treated and control groups. All data are plotted as means±SEM.

To assess the effect of IGFBP3 on myoblast differentiation, the C2C12 myoblasts were transfected at day 0 in differentiation media and cells were harvested every day from day 1 to 7. At day 3, we observed differentiation delays in Igfbp3AON treated cells compared to their counterparts treated with the control AON (Fig. 3D). The differentiation delay was most noticeable at day 5. The most likely explanation for the differentiation delay would be downregulation of IGFBP3 and related pathways. To assess this, first, RT-PCR was performed and the results showed almost 50% exon skipping 24h after the transfection, while this was hardly visible at 72h (Fig. 3A). Protein analysis at the given timepoints revealed the IGFBP3 downregulation in Igfbp3AON treated cells 2 days after the transfection. After day 2, IGFBP3 expression levels become almost equal in treated and control cells until day 6 (Fig. 3BC. After day 5, Igfbp3 AON treated cells displayed more efficient differentiation showing that the transient decrease of IGFBP3 caused a delay of differentiation but did not prevent it entirely.

### Differentially expressed genes in response to IGFBP3 downregulation

**Fig. 4:**
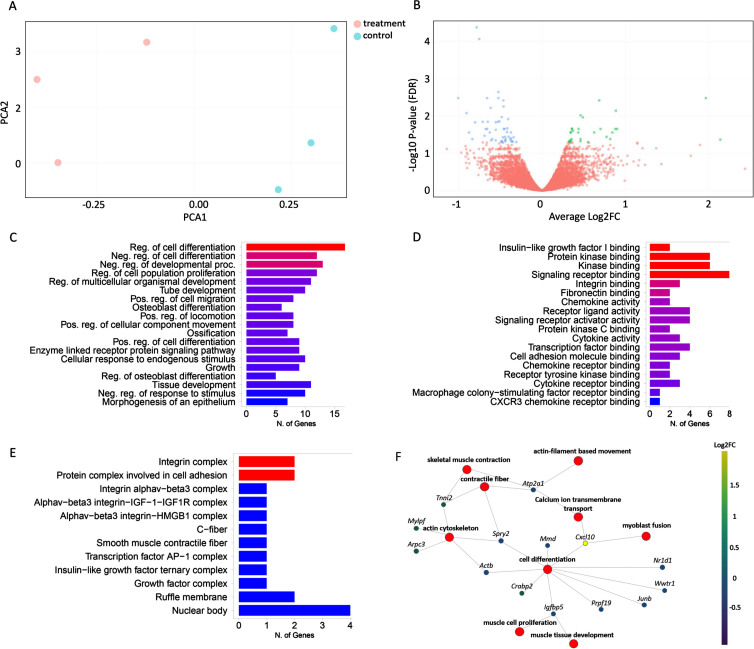
Effects of IGFBP3 downregulation on the related transcriptome and pathways. A. PCA plot showing the distance between treatment and control groups based on their similarities and differences. B. Volcano plot showing the difference in gene expression levels between treatment and control groups. The X-axis contains the Log2FC and the Y-axis shows -Log10 *P*-value. Down-regulated genes are shown on the left side of the plot, while up-regulated genes are shown on the right. The most statistically significant genes are placed at the top and shown in either blue or green. C. Bar graph showing the sorted list of the most enriched terms with the number of genes using the GO:biological process databases. D. Bar graph showing the sorted list of the most enriched GO molecular function terms. E. Bar graph showing the sorted list of the most enriched GO cell component analysis terms. F. The gene-concept network shows which genes are involved in the most significant terms. The most enriched terms along with all associated genes were collected and displayed in a network plot. The color given to genes is based on the log-fold change determined after the expression analysis. Ultimately, this plot shows the connection of genes between the most important road. The bar charts (C,D,E) are sorted based on *P*-value from top to bottom.

Having confirmed that IGFBP3 downregulation has a significant effect on the IGF-1 signaling pathway and thereby likely on myogenesis, we decided to further analyze the whole transcriptome in these cells by performing a 3’RNA sequencing experiment. When the raw data were obtained, the reads were aligned to the mouse reference genome (GRCm38/mm10) and about 82% of transcripts were found to be uniquely mapped. After filtering, DGE analysis was performed with 8464 genes by limma-voom [36], and edgeR tools [37]. Principal component analysis (PCA) revealed the clustering between the treated and control groups (Fig. 4A). Both tools reported around ∼150 DE genes between Igfbp3AON and control AON treated cells (92 genes downregulated, 51 genes upregulated) (Fig. 4B, Figure S3). This relatively low number of DE genes could be due to the fact that the C2C12 cells were transfected at the late stages of differentiation, providing limited room for change for most of the biological processes. GO annotations of the DE genes showed a strong enrichment for genes involved in regulation of developmental processes, signaling, cell communication, cell differentiation, muscle structure development and IGF-1 transport and signaling (Fig. 4C). Molecular function analysis mapped the majority of genes to protein binding and regulation of in particular acetylcholine receptor binding and regulation (Fig. 4D). GO cellular component ontology terms showed the subcellular structures and macromolecular complexes including IGF-1 and IGF-1R along with other cell signaling proteins and receptor complexes (Fig. 4E). The gene-concept network analysis showed that the most affected pathways in response to IGFBP3 downregulation were regulation of myogenesis (myoblast proliferation, fusion and differentiation), skeletal muscle contraction, regulation of actin cytoskeleton and positive regulation of calcium ion transmembrane transport (Fig. 4F).

For the myogenic pathways, 29 genes were downregulated and 8 genes were upregulated in response to AON treatment (*Neat1, Ccl2, Arrdc3* and *CxCl10* and *Apol9a* being most significantly affected respectively) (Table 3). Calcium homeostasis was affected by the downregulation of *Atp2a1* and *Atp2a2* genes. As expected, IGF-1 signaling, by means of transport and uptake, was also affected upon IGFBP3 downregulation. Among the genes involved in this process, *Igfbp5*, another binding protein of IGF-1, was found to be downregulated. Furthermore, IGFBP3 downregulation had an impact on apoptotic pathways by affecting the number of related genes (Table 3) presumably due to IGF-1 independent actions of IGFBP3 [38].

**Table 3:** Pathways affected significantly in response to IGFBP3 downregulation

Enriched term	*P*-value	Downregulated genes	Upregulated genes
Regulation of cell differentiation	1.42^E^-05	*Junb,Npnt,Wwtr1,Prpf19,Kctd11,Spry2,Csf1,Igfbp5, Nr1d1,Bin1,Ywhag,Actb,Itgav,Sema7a,Sox9,Wdr1,Trim72*	*Cxcl10,Crabp2*
Muscle structure development	6.17^E^-05	*Npnt,Ly6e,Igfbp5,Bin1,Cdh2,Myom2,Actb,Atp2a2,Sox9, Wdr1,Trim72*	*Cxcl10,Mylpf*
Regulation of intracellular signal transduction	2.21^E^-04	*Ccl2,Hexim1,Npnt,Arrdc3,Wwtr1,Dusp6,Spry2,Csf1,Igfbp,Nr1d1,Psap,Cdh2,Itgav,Sema7a,Sox9,Ddx5,Tob1*	*Gadd45g*
Muscle cell differentiation	2.70^E^-04	*Npnt,Igfbp5,Bin1,Cdh2,Myom2,Atp2a2,Sox9,Wdr1,Trim72*	*Cxcl10*
Muscle system process	2.84^E^-03	*Atp2a1,Npnt,Igfbp5,Bin1,Myom2,Atp2a2,Trim72*	*Mylpf,Tnni2*
Cell death	4.50^E^-03	*Ccl2,Atp2a1,Dusp6,Angptl4,Spry2,Csf1,Psap,Phlda1,Bin1, Actb,Itgav,Atp2a2,Sox9,Ddx5*	*Cxcl10,Gadd45g, Xpa,Atpif1,Pea15a*
Acetylcholine receptor signaling pathway	6.73^E^-03	*Ly6a,Ly6e,Ly6c2, Ly6c1*
MAPK cascade	1.16^E^-02	*Ccl2,Npnt,Dusp6,Spry2,Psap,Cdh2,Itgav,Sema7a,Sox9*	*Gadd45g,Pea15a*
Regulation of cell population proliferation	1.53^E^-02	*Ccl2,Junb,Wwtr1,Kctd11,Spry2,Csf1,Igfbp5,Nr1d1,Cdh2, Glul,Actb,Itgav,Sox9,Tob1*	*Cxcl10,Atpif1*
Apoptotic process	1.78^E^-02	*Ccl2,Atp2a1,Dusp6,Angptl4,Spry2,Phlda1,Bin1,Itgav, Sox9,Ddx5*	*Cxcl10,Atpif1, Pea15a*
Muscle contraction	3.17^E^-02	*Atp2a1,Npnt,Bin1,Myom2,Atp2a2*	*Mylpf,Tnni2*
Positive regulation of endoplasmic reticulum calcium ion concentration	3.96^E^-02	*Atp2a1,Atp2a2*
Reduction of cytosolic Ca++ levels	5.31^E^-03	*Atp2a1,Atp2a2*
Regulation of Insulin-like Growth Factor (IGF) transport and uptake by Insulin-like Growth Factor Binding Proteins (IGFBPs)	3.54^E^-03	*Csf1,Igfbp5,Cdh2, H19*	*Apol9a*
RAF/MAP kinase cascade	4.09^E^-03	*Dusp6,Actb*	*Pea15a*

Table 3. Pathways affected significantly in response to IGFBP3 downregulation

### The AONs were not able to function in vivo

**Fig. 5:**
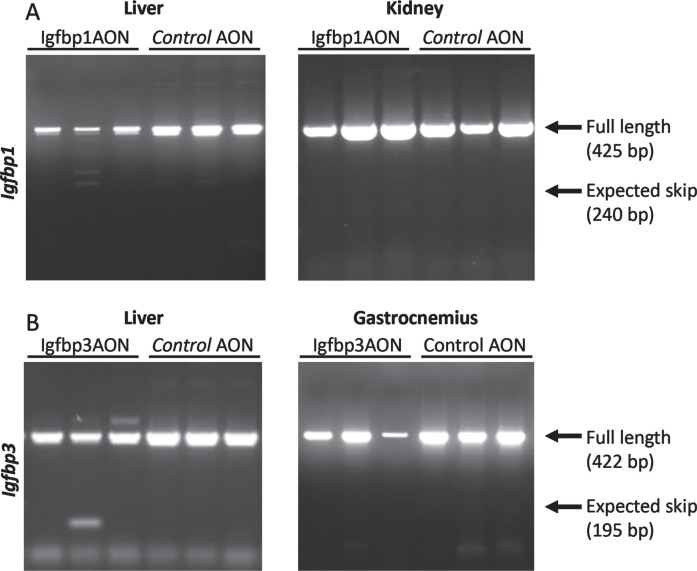
RT-PCR assessment of exon 2 skipping in *Igfbp1* (A) and *Igfbp3* (B). The arrows on the gel correspond to full length (non-skipped) and expected skip PCR products. No skipped products were detected.

After obtaining proof-of-concept *in vitro*, the AONs were further tested *in vivo*. Here, three *mdx* males were treated twice weekly with Igfbp1 (20mg/kg) and Igfbp3 (40mg/kg), or control AONs for 6 weeks. Mice were sacrificed four days after the final injection and tissues were isolated for RNA and protein analysis. These doses were however insufficient to induce exon 2 skipping in the gastrocnemius or liver for any of the targets (data not shown). To increase treatment efficacy, we increased the AON dose and doubled the number of injections per week. Exon 2 skipping levels were determined in the gastrocnemius, liver, and kidney. Unfortunately, despite the altered treatment regime, AONs did not induce exon 2 skipping *in vivo* (Fig. 5).

**Fig. 6:**
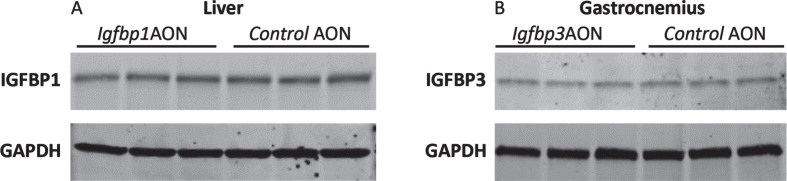
Western blot analysis of IGFBP1 and IGFBP3. A. Blot picture showing the IGFBP1 levels in Igfbp1AON treated cells compared to control AON treated cells. B. Blot picture showing the IGFBP3 levels in Igfbp3AON treated cells compared to control AON treated cells. GAPDH was used as loading control.

To assess potential effects on protein level, Western blotting analysis was performed for IGFBP1 and IGFBP3 using protein isolates from the liver and gastrocnemius. No reduction was observed for IGFBP1 or IGFBP3 protein levels in cells treated with exon skipping AONs compared to the controls (Fig. 6). Taken together, the AONs were not able to function in *mdx* mice even though they efficiently induced exon skipping and protein knockdown *in vitro*. No significant difference was observed in serum markers for liver and kidney function and damage (ALP, GTP, GOT, Urea) in the plasma of *mdx* mice treated with IgfbpAONs and control AONs (Figure S4).

## DISCUSSION

It has been shown feasible to restore dystrophin expression in DMD patients using gene and genetic therapies. However, these approaches restore partially functional dystrophin proteins (exon skipping and gene therapy) or very low levels of full-length dystrophin (stop codon readthrough). Therefore, it is likely that maximal benefit will be achieved by a combination of dystrophin restoration and improvement of the secondary pathology. Manipulation of specific target proteins involved in inflammation, fibrosis or muscle regeneration could be a good strategy to improve muscle quality and to slow down disease progression. Many research groups have focused on using IGF-1 as a therapeutic option in neuromuscular diseases including DMD, given that IGF-1 has been shown to regulate muscle growth and function [39]. To boost IGF-1 levels, it was first thought that the most straightforward method would be systemic administration of IGF-1 peptides. The first obstacle of this approach was the fact that systemically injected IGF-1 is rapidly captured by IGFBPs excessively found in blood [40]. Additionally, the very short half-life of unbound IGF-1 peptide made this approach even more challenging [41]. Progress has been made on both allowing IGF-1 to escape binding to IGFBPs and improving its half-life, but so far, desired functional improvements in clinical studies have not been obtained [15].

IGF-1 is mostly produced in liver, but there is also local expression in skeletal muscle, which gives rise to different splice isoforms with unique functions [42]. More recently, a clinical trial showed that the rhIGF-1 treatment in DMD boys improved the linear growth but did not change the motor function. This highlights the significance of the route of administration and locally produced IGF-1 in skeletal muscle. To make IGF-1 treatment muscle specific, viral-mediated gene transfer and tissue-specific overexpression approaches have been studied and more prominent results have been obtained in animal models. However, clinical translation will be challenging, as currently only adeno associated viral vectors can efficiently deliver genetic cargos to skeletal muscle and thus using viral-mediated gene transfer to overexpress IGF-1 precludes patients from receiving micro-dystrophin. In addition to that, continuous expression of IGF-1 may not be desired in myogenesis where everything is dynamic from satellite cell activation to myofiber formation [43]. Therefore, other components of the IGF-1 system such as IGFBPs could be potential targets. In the present study, our aim was to enhance IGF-1 signaling *in vitro* and *in vivo* by downregulating its binding proteins, IGFBP1 and IGFBP3, using exon skipping AONs.

Here, we downregulated IGFBP1 and IGFBP3 and assessed their effects on IGF-1 in both an endocrine and autocrine/paracrine manner respectively. Because IGFBP1 is expressed in hepatic tissues but not in skeletal muscle, when downregulated, it would affect IGF-1 signaling in an endocrine manner only by increasing the free IGF-1 level in circulation. However, IGFBP3 is expressed in both hepatic and extrahepatic tissues including skeletal muscle. When IGFBP3 is downregulated, it would increase the free IGF-1 level not only in circulation but also in skeletal muscle where IGFBP3 and IGF-1 are locally expressed. Furthermore IGFBP3-IGF-1 complexes in circulation make an additional binding with ALS protein forming a huge complex which is not able to exit the circulation unless cleaved by proteases.

Our results showed that AON-mediated downregulation of IGFBP1 and IGFBP3 proteins led to enhanced IGF-1 signaling *in vitro* confirmed by increased Akt phosphorylation. However, the encouraging results we observed *in vitro*, were not translated into the *in vivo* study where we subcutaneously injected *mdx* mice for 6 weeks. After self-dimerization and degradation possibilities of the AONs were ruled out (Figure S2), the AON concentrations were increased (approximately two times) and the number of injections were doubled, for the remaining of the mice (*n* = 3) to be treated. Despite these experimental changes, no exon skipping was observed.

Delivery of AONs to the target cells in the target tissues in a functional compartment is one of the major challenges. Because liver is the major site of PS-modified AON accumulation [44] and the main source of IGFBP1 expression [45], very efficient exon skipping was expected, as most AONs are cleared by liver and kidney. However, PS AONs accumulate in non-parenchymal cells (Kupffer cells, liver sinusoidal endothelial cells, hepatic stellate cells, and other cell types) where IGFBP1 is not being expressed [46, 47]. This could be one of the explanations of the AON ineffectiveness in the *mdx* mice. IGFBP3 in skeletal muscle is mainly expressed in macrophages where AON concentrations are expected to be higher [47, 48], and uptake is likely higher due to their pronounced phagocytic activity [49]. However, it has been shown that the PS-AONs in macrophages are sequestered in vacuoles due to cytokine secretion resulting in slow release of the AONs into the cells and nuclei. This could be one of the limiting factors for inefficient *Igfbp3* exon skipping in the skeletal muscles of *mdx* mice [49].

Instead of AONs, downregulation of a target gene on RNA level could also be achieved by the use of small interfering RNAs (siRNA). Here, treatment efficacy will also depend on successful delivery. siRNAs with GalNac conjugates have been successfully delivered to liver hepatocytes where most target proteins are expressed [50]. Other chemical modifications such as cholesterol conjugates on siRNA now allow efficient uptake by skeletal muscle [51]. Notably, these conjugates will also improve delivery of AONs and could be useful for IGFBP1 and IGFBP3 knockdown in liver and skeletal muscle in future developments.

IGFBP3 is known to have antiproliferative and differentiation promoting effect in human primary myoblasts [35]. When IGFBP3 was downregulated prior to myogenic differentiation, it resulted in a differentiation delay in C2C12 myoblasts. It should be noted that the C2C12 myoblasts were transfected at day 0 in differentiation media with a lower amount of transfection reagent to avoid toxicity. Furthermore, cells were at 70% confluency and some will have divided after transfection, resulting in a dilution of AON levels. These aspects could be the reason of the quick recovery of IGFBP3 levels at day 3 in differentiation media. Nevertheless, even with a low level of IGFBP3 downregulation, a remarkable delay was obtained at the end of normal differentiation duration (day 7). Taken together, these data indicate that IGFBP3 promotes myoblast differentiation but further experiments, such as myoblast proliferation assay and immunostaining for myotube analysis, should be conducted to draw a more comprehensive conclusion regarding the effect of IGFBP3 downregulation on myoblast proliferation and differentiation.

In IGFBP3 downregulated C2C12 cells, 3’RNA sequencing unraveled the DE genes involved in myogenic pathways such as cell proliferation, fusion and differentiation. *Neat1*, a long noncoding RNA, which accelerates myoblast proliferation and suppresses cell differentiation [52], was significantly downregulated in response to IGFBP3 downregulation. The reduction of *Neat1* could be due to the increased free-IGF-1 levels in IGFBP3 downregulated cells that would promote cell differentiation. *Hmga1*, a myogenic marker that suppresses myoblast differentiation, was downregulated as well [53]. The ERK-1/2 mediated signaling pathway is one of the major components of IGF-1 downstream signaling and promotes proliferative pathways in skeletal muscle [54].

CXCL10 is a chemokine which was shown to enhance muscle regeneration by promoting myoblast differentiation [55]. We found that *Cxcl10* expression was significantly upregulated in response to IGFBP3 downregulation. Further, *Apol9a* expression is known to increase along with myoblast differentiation by regulating the ERK1/2 pathway.

The 3’RNA-seq results revealed that *Apol9a* expression is significantly upregulated in cells treated with Igfbp3AON. On the other hand, *Junb, Ctgf* and *Hexim1*, which would normally promote cell differentiation, were found to be downregulated in response to IGFBP3 knockdown, but their effect was statistically less significant. This could be due to IGF-1 independent roles of IGFBP3 on cell proliferation given that IGFBP-3 has various IGF-independent actions including inhibition of proliferation, survival and migration as well as modulation of angiogenesis [56]. Interestingly, *Igfbp5* expression was found to be downregulated. IGFBP5, one of the binding proteins of IGF-1, plays a role in myogenesis by promoting cell differentiation but IGFBP5 can also act independently of IGF-1 by activating a distinct receptor so that its downregulation might have other effects on biological processes [57].

RNA therapeutics that are efficiently delivered could pave the way for enhanced IGF-1 signaling in skeletal muscle and other tissues by downregulating liver-derived and/or locally expressed IGFBPs. Our proof-of-concept study revealed the feasibility of this approach as well as its effects on the related transcriptome. The results also point out the ineffectiveness of unconjugated AONs for protein knockdown even in the liver, and the need of conjugates to ensure efficient delivery.

## Supplementary Material

Supplementary Material

## Data Availability

The data supporting the findings of this study are openly available in NCBI BioProject database (PRJNA973858) and within the supplementary material.
