# Supplementary material for: Antisense Oligonucleotide-Mediated Downregulation of IGFBPs Enhances IGF-1 Signaling
Source: J Neuromuscul Dis. 2024 Mar 5;11(2):299–314. doi: 10.3233/JND-230118 (PMC10977368; doi:10.3233/JND-230118)
Supplement: Supplementary Material [file jnd-11-jnd230118-s001.docx]

**Supplementary data**


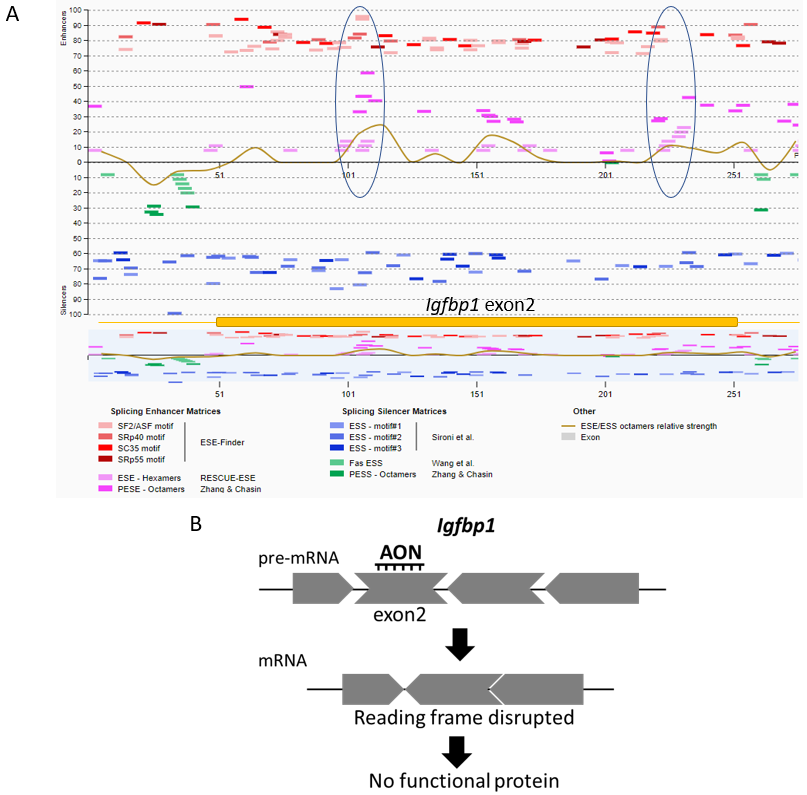


**Figure S1.** ESE motifs on exon 2 of *Igfbp1* pre-mRNA as representative example of AON design.


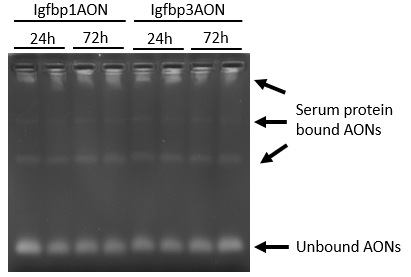


**Figure S2.** Gel picture showing the self-dimerization and degradation test results for Igfbp1 and Igfbp3 AONs treated with mouse serum for 24 or 72h. The bands on the gel show AON/protein complexes and unbound AONs.


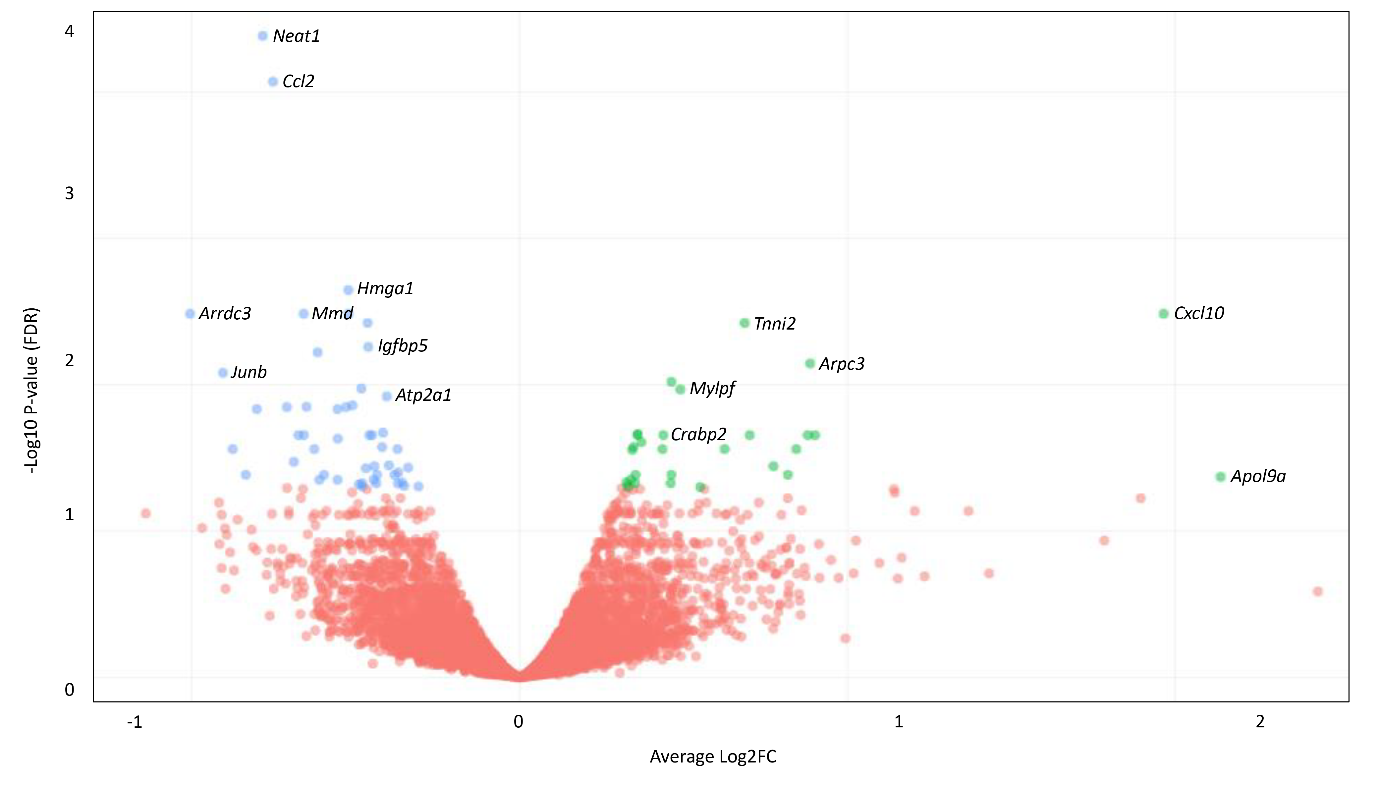


**Figure S3.** The Volcano plot showing the differentially expressed genes related to myogenic pathways; blue dots represent the downregulated genes, green dots represents the upregulated genes


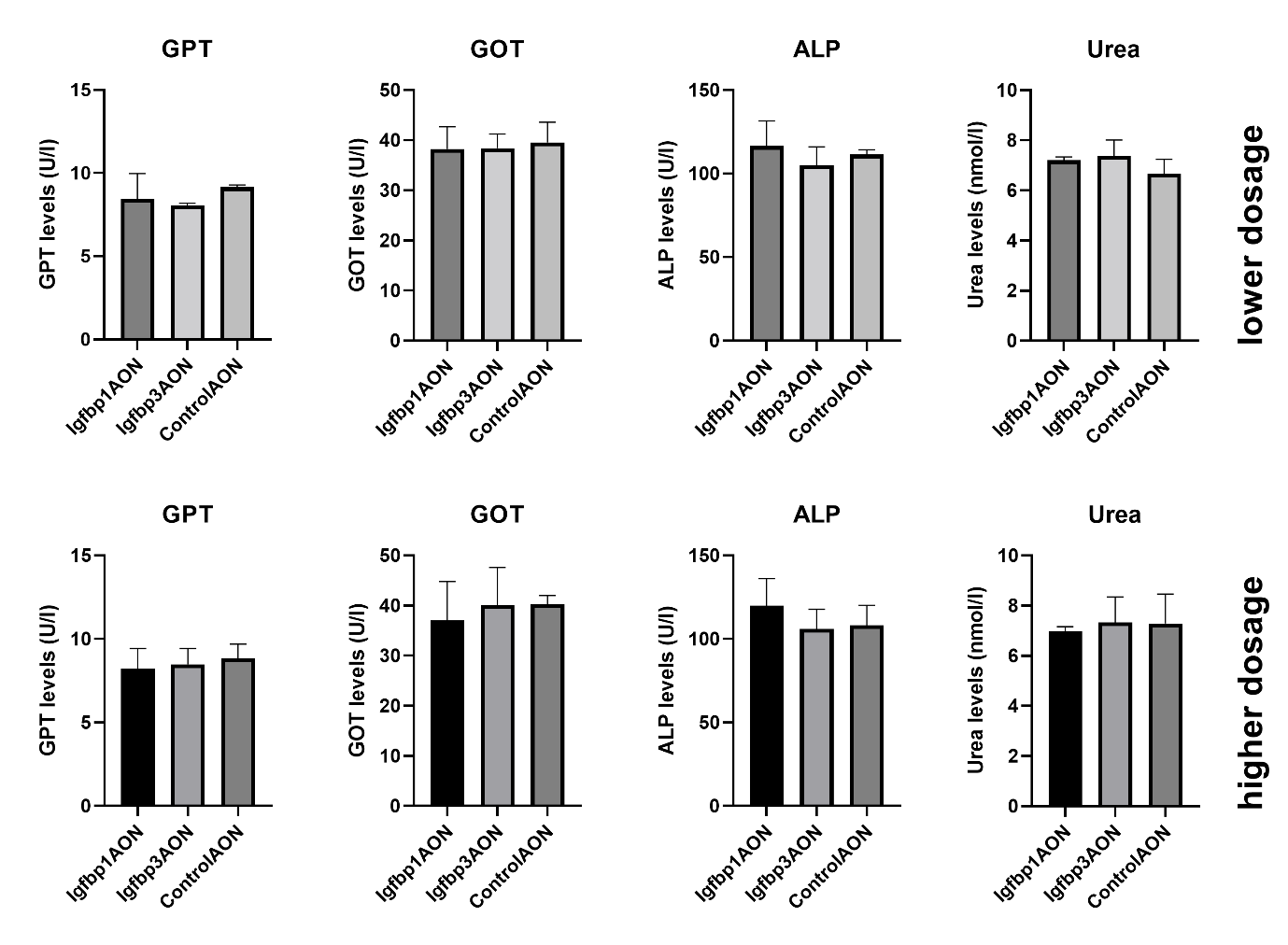


**Figure S4.** The tolerability of the Igfbp1 and Igfbp3 AON treatment was assessed by quantification of markers for kidney and liver function and damage in wildtype mice (n=6 per group). ALP: alkaline phosphatase, GOT: glutamic oxaloacetic transaminase, GPT: glutamic-pyruvic transaminase. Lower dosage; 40 mg/kg two times per week. Higher dosages; 50 mg/kg (Igfbp1AON) and 60 mg/kg (Igfbp3AON) four times per week during six weeks.
